# Aging engineers’ occupational self-efficacy—a mixed methods study

**DOI:** 10.3389/fpsyg.2023.1152310

**Published:** 2023-05-18

**Authors:** Stina Wallin, Anncristine Fjellman-Wiklund, Lisbeth Fagerström

**Affiliations:** ^1^Faculty of Education and Welfare Studies, Health Sciences, Åbo Akademi University, Vaasa, Finland; ^2^Department of Community Medicine and Rehabilitation, Physiotherapy, Umeå University, Umeå, Västerbotten, Sweden; ^3^Faculty of Health and Social Sciences, University of South-Eastern Norway, Drammen, Norway

**Keywords:** occupational self-efficacy, personal resources, aging workers, engineers, full working life, mixed methods

## Abstract

**Introduction:**

Engineers’ work has become more complex with increased demands in today’s changing working life. Self-efficacy is essential to successfully adapt to work-related changes and to cope with adverse job demands. However, less is known about aging engineers’ occupational self-efficacy. Therefore, this study explores facilitators and barriers to aging engineers’ occupational self-efficacy beliefs to continue working until expected retirement age. An additional purpose is to explore if any of the aspects described by the engineers are more prominent.

**Methods:**

The study design was exploratory, using mixed methods with a qualitative to quantitative approach. A total of 125 engineers, aged between 45 and 65 years, answered two open-ended survey questions about what positively and negatively affect their occupational self-efficacy beliefs to continue working. First, data was analyzed using an inductive manifest qualitative content analysis. Next, descriptive statistics were performed based on the results of the qualitative study.

**Results:**

The analyses revealed that health and working conditions that affect health were crucial facilitators and barriers for the aging engineers’ occupational self-efficacy to continue working until expected retirement age. Furthermore, the engineers emphasized competence, motivation from meaningful tasks, family and leisure, and private economy.

**Discussion:**

The aging engineers’ own health seems to be prominent in their self-efficacy regarding a full working life; consequently, support still needs to address issues affecting health.

## 1. Introduction

Retaining aging workers longer in working life has been a much-discussed topic during recent years (Eurofound, 2021), and is an issue that also applies to engineers. The need for engineers is increasing because of the fast and continuous technical development (Cedefop, 2020). In Finland, there is also a high number of retiring engineers. To meet these needs, it is important to retain aging engineers in working life, also. However, working life today is continuously changing with increasing work pressure, demands and instability [International Labour Office (ILO), 2019]. Engineers’ work has changed as well. It has become more complex, interconnected, and interdependent, and requires for instance global competence, unique technical skills, creativity, and social and entrepreneurial skills (Qadir et al., 2020). One concept that seems to be essential for adaption to changes (Turgut and Neuhaus, 2020), and important in working life (Bakker and Demerouti, 2017) is occupational self-efficacy. Nevertheless, occupational self-efficacy is less studied among engineers (Chen et al., 2023) and aging workers (Paggi and Jopp, 2015; Chiesa et al., 2016). Exploring what influence aging engineers’ occupational self-efficacy can reveal ways to support them for longer working life.

## 2. Background

Occupational self-efficacy is a personal resource defined as the belief in one’s own capabilities to successfully perform activities involved in one’s work (Rigotti et al., 2008), and is based on the concept developed by Bandura (1997). According to Bandura, people influence their thoughts, feelings, motivation, and actions (Bandura, 1997; Bandura, 2012). Self-efficacy does not refer to the number of skills one has, but to what one believes one can do with whatever skills one holds under different circumstances (Bandura, 1997). Individuals with high self-efficacy choose more challenging tasks, set more ambitious goals, motivate themselves, expend more effort to reach their goals, and persist longer when facing difficulties (Bandura, 1997; Bandura, 2012). Self-efficacy beliefs are developed in four ways: previous successful experiences, vicarious experience by observing peers succeed, verbal persuasion from significant others, and perceived physiological and emotional states. Sustainable and resilient self-efficacy requires experiences of overcoming obstacles through persistent effort (Bandura, 1997; Bandura, 2012).

Occupational self-efficacy is important in working life (Fullemann et al., 2015; Paggi and Jopp, 2015). Previous research has reported positive relationships between occupational self-efficacy and, for example, work motivation, job performance (Cetin and Askun, 2018), work engagement (Guarnaccia et al., 2018; Liu, 2019), job satisfaction and health (Guarnaccia et al., 2018). People with high occupational self-efficacy have reported higher well-being at work (Bakker and Demerouti, 2017), despite high job demands (Onyishi et al., 2018). Tomas (2021) suggested that by increasing occupational self-efficacy, perceived mental strain might decrease and individual and organizational well-being might be promoted. However, Rigotti et al. (2018) found in their study that high job demands, and high strain negatively affected the relationship between career-related self-efficacy and career satisfaction. Other studies have considered that self-efficacy is mentally protective against challenging situations, stress, and burnout (Bandura, 2012; Shoji et al., 2016; Schönfeld et al., 2017). Some studies were found concerning aging workers, in which positive results of occupational self-efficacy on job satisfaction (Paggi and Jopp, 2015; Wöhrmann et al., 2017), life satisfaction, work motivation (Paggi and Jopp, 2015), and health (Wöhrmann et al., 2017) were reported.

Few studies were found regarding occupational self-efficacy among engineers, and aging engineers, in particular. A recent study highlighted that research regarding self-efficacy of engineers is often conducted in the context of science, technology, engineering, and math (STEM), and that such research often generalizes the subjects of one STEM discipline to those of others (Chen et al., 2023). A meta-analysis evaluated the effect of the sources of self-efficacy (mastery experiences, vicarious learning, verbal persuasion, and affective states) on outcome expectations in STEM disciplines. The findings showed strongest effect for the three first-mentioned sources (Sheu et al., 2018). In a study that qualitatively investigated the sources of undergraduate engineering students’ self-efficacy to complete engineering tasks, the most relevant sources were mastery experiences through academic performance and emotional experiences, as well as vicarious experiences through social interactions and encouragement from instructors and engineers (Chen et al., 2023). Chen et al. (2023) highlighted the scarce of studies regarding engineers’ experiences of what creates their work-related self-efficacy. Panatik et al. (2011) found that general self-efficacy decreased the negative effects of job demands on psychological strain among technical workers, 22–55 years of age. However, these studies included mainly younger persons. A recent study that included aging engineers, aged 45 and older, showed that higher occupational self-efficacy was more often related to a good work ability (Wallin et al., 2021), referred to as the balance between individual resources and job demands (Ilmarinen, 2012). These findings indicated that enhancing occupational self-efficacy might help aging engineers to cope with job demands, as well as support their work ability (Wallin et al., 2021). Similarly, alongside job resources, personal resources have shown to reduce the negative effects of age on work ability (Converso et al., 2018). Work ability is, beside health, an important marker for longer working lives (Jääskeläinen et al., 2016; Laaksonen et al., 2022). Although previous research indicates positive effects of occupational self-efficacy on engineers’ working life, there are few studies regarding aging engineers and especially about what generates their occupational self-efficacy for a longer working life.

A theoretical model that considers the importance of personal resources, such as self-efficacy, alongside with job demands and job resources is the well-known Job Demands-Resources theory (J D-R; Bakker and Demerouti, 2017; Bakker and Demerouti, 2018). In this theory, job demands are “those physical, psychological, social, or organizational aspects of the job that require sustained physical and/or psychological effort” that are associated with physiological and/or psychological costs (Bakker and Demerouti, 2017, p.274). High job demands are associated with a health-impairment process, presumed to weaken health (Bakker and Demerouti, 2018). However, positively valued job demands do not affect well-being negatively when job resources are enough (Bakker et al., 2010). Job resources, on their part, are physical, psychological, social, or organizational aspects that either reduce job demands and the associated physiological and psychological costs, or have a motivational potential in achieving work goals, or stimulating personal growth, learning and development (Bakker and Demerouti, 2017; Bakker and Demerouti, 2018). Personal resources, such as self-efficacy, are perceived to play similar roles as job resources (Bakker and Demerouti, 2017; Bakker and Demerouti, 2018). There is also a reversed effect that job resources improve personal resources, which in their turn improve job resources (Bakker and Demerouti, 2018). In J D-R theory, personal resources refer to individuals’ sense of whether they can control and influence their environment successfully (Hobfoll et al., 2003; Bakker and Demerouti, 2017). Although persons with high personal resources do not perceive fewer job demands they are more resistant to adverse demands by dealing with them in an active and effective way (Bakker and Demerouti, 2017). Thus, personal resources such as occupational self-efficacy is expected to support aging engineers against adverse job demands as well.

Furthermore, practices for supporting aging workers seldom focus on increasing personal resources; they mainly center on decreasing job demands and increasing job resources (Cloostermans et al., 2015). Scholars have recommended to include strengthening of occupational self-efficacy in human resource programs (Guarnaccia et al., 2018) and career development programs (Turgut and Neuhaus, 2020). Cetin and Askun (2018) highlighted that motivation-related concerns are often pointed out when an employee is underperforming. They suggested instead that self-efficacy doubts should be investigated before making motivation related judgments, and that supporting self-efficacy should be included in employee development programs. For a sustainable working life, the individual needs of the workers must be considered (Eurofound, 2022).

Based on the existing research, the importance of occupational self-efficacy is confirmed. However, aging engineers’ occupational self-efficacy seems to be under-researched. We assume that by enhancing occupational self-efficacy, aging engineers can be supported to meet current increasing and changing demands in working life. To be able to support aging engineers’ working life, knowledge about what aging engineers consider important for their occupational self-efficacy beliefs need to be explored.

## 3. Purpose

The aim of this study is to explore facilitators and barriers to aging engineers’ occupational self-efficacy beliefs to continue working until expected retirement age.

The research questions are:

What aspects positively and negatively affect aging engineers’ occupational self-efficacy to continue working until expected retirement age?
How are the emerged categories distributed, based on the number of times every aspect mentioned by each participant appeared within their category?

## 4. Materials and methods

### 4.1. Study design, participants, and data collection

This study had an exploratory design using a mixed methods light with a qualitative to quantitative approach (Creswell and Plano Clark, 2018; Kyngäs et al., 2020). The study is part of a research project, “Occupational self-efficacy supporting a full working life,” that includes aging workers from engineering and home care sectors (Wallin et al., 2020, 2021, 2022). We considered aging workers 45 years and older. Forty-five years is an often-used criterion for aging workers based upon those major changes in function frequently occurring at this age and that can affect work ability and personal resources; yet preventive measures are still possible at this stage (Ilmarinen, 2001). Forty-five years and older engineers were invited by purposive sampling to answer an anonymous pilot tested web-based questionnaire available in Swedish and Finnish. First, in order to meet the inclusion criteria, that is, engineers, 45 years and older, and having a valid employment contract, discussions took place with the head of the human resource management in six globally productive companies in Finland. Next, engineers fulfilling the inclusion criteria were invited. Engineers from two companies were excluded before the survey was sent out, because they did not meet the inclusion criteria of the minimum age of 45. Thus, engineers from four companies were finally included. Each company’s local human resource department provided participants with the study information letter and the link to the web-based questionnaire. The anonymous questionnaires were returned to the site for questionnaires (E-lomake) at Åbo Akademi University’s server, which is secured by personal username and password. The data was thereafter exported to Excel, where the data analyses were performed. The exported survey responses were initially checked for duplicates and whether participants met inclusion criteria. The period to collect data was set for May to June in 2018 and was extended to September to enable a larger participation. The data collection ended at the end of September, despite some difficulties in recruiting.

The questionnaire included four open-ended questions and three reputable, valid, and reliable measuring scales, which were analyzed separately in different phases. The results from the measuring scales Work Ability Index (Tuomi et al., 1998), Utrecht Work Engagement Scale (Schaufeli et al., 2006) and Occupational Self-Efficacy Scale—Short Form (Rigotti et al., 2008) are presented elsewhere (Wallin et al., 2021).

**Figure 1:**
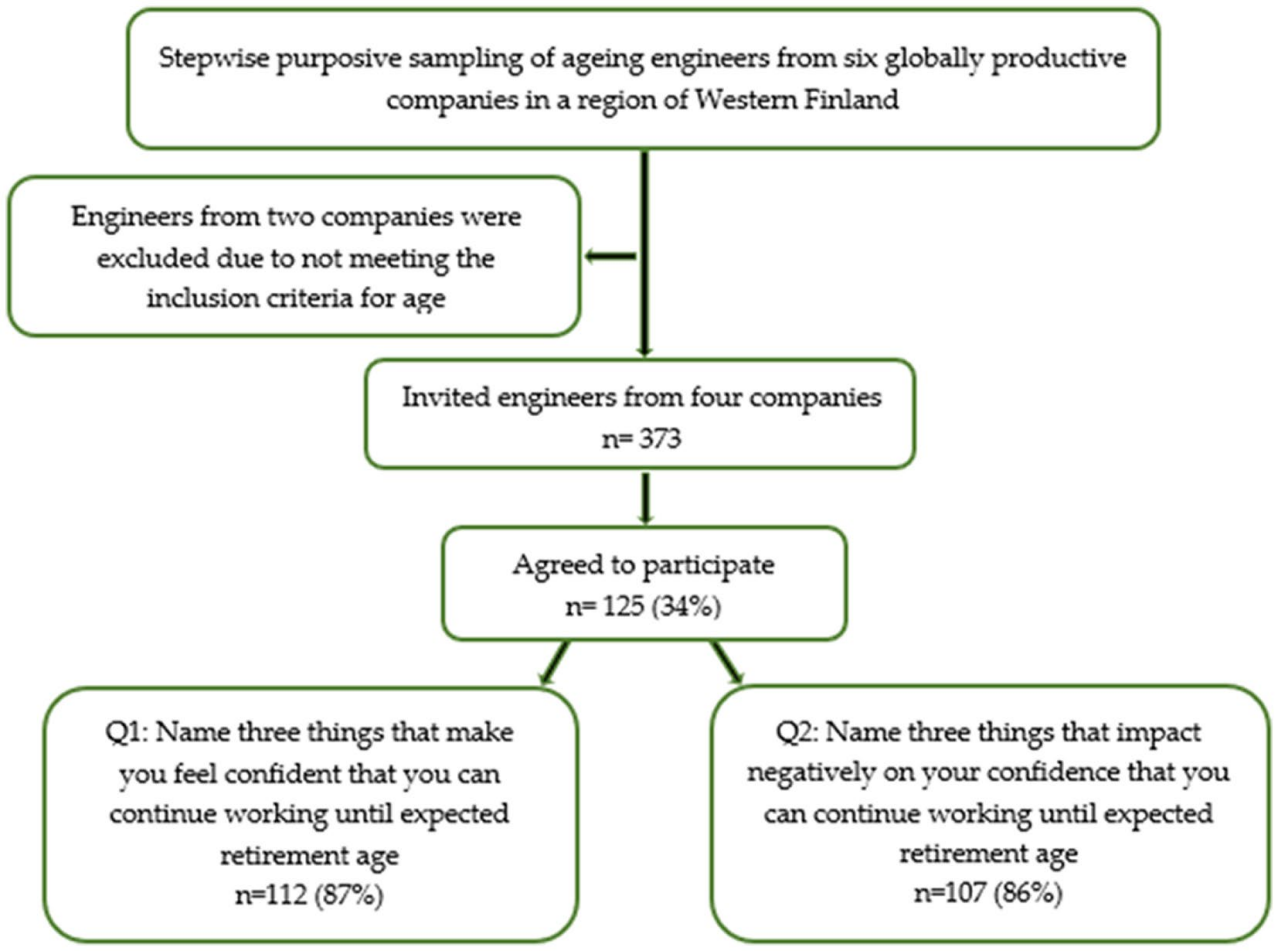
Flowchart of sample selection and the study population.

The answers from two open-ended questions constituted the source of this study. The engineers were asked to name three things that make them feel confident that they can continue working until expected retirement age and correspondingly what impact negatively on their confidence to continue working (i.e., occupational self-efficacy; Figure 1). The open-ended questions were articulated based on the scarce of studies that have explored aging engineers’ experiences about what generates their occupational self-efficacy for a full working life. Furthermore, Eurofound (2022) has highlighted the significance of individual needs for sustainable working life.

Out of 373 invited participants, 125 engineers (34%) answered the questionnaire after three reminders. The participants were between 45 and 65 years old, and the majority were men (n = 109). The socio-demographic characteristics of participants are shown in Table 1.

**Table 1:** Characteristics of the study population (*n* = 125).

**Age (years)**	**53 (52), 5.1**
45–49	33.3
50–54	32.5
55–59	22.8
60–66	11.4
**Gender**	
Women	12.1
Men	87.9
**Marital status**	
Single	7.2
Married/marriage-like relationship	84.8
Divorced	8.0
Widow	0.0
**Native language**	
Swedish	28.0
Finnish	72.0
**Educational level**	
Vocational degree	0.8
Higher vocational education	72.6
Master’s degree or higher education	26.6
**Employment status**	
Permanent employment	97.6
Temporary employment	2.4
**Work ability in relation to job demands (rather good-very good)**	
In relation to mental demands	77.0
In relation to physical demands	85.0
Work ability index	41 (43), 6.0
Work ability score	8.3 (9.0), 1.5
Occupational self-efficacy	5.8 (6.0), 0.8
Work engagement	4.5 (5.0), 1.3
Work experience (years)	18 (20), 9.7

The numbers are either % or mean (median), standard deviation. Work ability index: poor (7–27), moderate (28–36), good (37–43), and excellent (44–49) work ability. Work ability score: current work ability compared with lifetime best 0–10, higher scores indicate better work ability. Work ability in relation to physical and mental demands: very poor-very good, % rather good-very good. Occupational self-efficacy: 0–7, higher mean scores indicate better self-efficacy. Work engagement: 0–6, higher mean scores indicate better work engagement.

### 4.2. Data analysis

A mixed methods approach was used, since integrating both qualitative and quantitative analyses to generate new knowledge enables a better understanding of a complex topic (Regnault et al., 2018; Kyngäs et al., 2020). In this study, data analysis and integration were conducted by transforming the qualitative categories into quantitative counts inspired by Creswell and Plano Clark (2018). First, the answers from the open-ended questions were manually analyzed by an inductive qualitative manifest content analysis, to gain a deeper knowledge and understanding (Graneheim and Lundman, 2004; Graneheim et al., 2017; Kyngäs et al., 2020) of the engineers’ occupational self-efficacy beliefs. The answers were organized in Microsoft Excel, with word-for-word answers from each participant in a separate row. All responses were read through several times to enable familiarization with the text and to obtain a sense of the whole. Next, responses from each question were grouped into content areas, which were analyzed by manifest qualitative content analysis. The condensed text was coded, and the codes were interpreted and repeatedly compared for similarities and differences in a forth and back process. Codes with similar content were sorted into sub-categories. Finally, sub-categories were sorted into categories that corresponded to the meaning of the material, the context, and the aim (Graneheim and Lundman, 2004; Graneheim et al., 2017).

The researchers’ pre-understanding consisted of experiences in qualitative and quantitative research, as well as long experience in health care, occupational health service and rehabilitation. To minimize bias in the pre-understanding, the qualitative data analysis followed a structured procedure (Graneheim and Lundman, 2004; Graneheim et al., 2017; Kyngäs et al., 2020). To confirm trustworthiness and conformability, investigator triangulation was used. The first author made the initial analysis of the responses into similar content areas, condensed, and coded the text. Thereafter, the co-authors read the condensed answers and the codes independently. Through a process of comparison and discussion on differences the researchers agreed on the codes and categories. To avoid bias because of the often short answers in the open-ended questions, the authors returned repeatedly to the meaning units as well as all the participants’ answers to check the entirety and the meaning. Furthermore, dependability was established by repeatedly going back to the encoding, verifying the encoding against the meaning units and the open-ended answers, and checking the reliability of the emerged categories (Graneheim and Lundman, 2004; Graneheim et al., 2017; Kyngäs et al., 2020). Notes about the encoding were made throughout the analysis process. Quotations were applied to support authenticity in the emerged categories (Table 2; Kyngäs et al., 2020).

**Table 2:** Results of the inductive manifest qualitative content analysis.

Categories positively affecting occupational self-efficacy to continue working until expected retirement age		
Categories	Sub-categories	Quotations
Engagement in one’s own health	Satisfactory physical and mental health	*“One’s own state of health”*
		*“I am physically and mentally in very good shape”*
	Health supporting activities	*“I take care of myself”*
		*“Notices more disabled workmates”*
		*“Opportunities to reduce the quantity of work to adapt it to [one’s own] work ability”*
Confidence in one’s own competence	Obtained knowledge and acquired skills	*“Perspective that experience (=age) has brought”*
		*“I possess knowledge that not many people have”*
		*“That the competence one has is valued”*
		*“I feel that I still keep up with today’s requirements very well”*
	Ambition and potential for development	*“I want to learn new things”*
		*“To channel my creativity”*
		*“Still able to learn new things”*
Healthy work environment	Adequate workload	*”The work is not that physically [demanding]”*
		*“Suitable workload (also mentally)”*
	Flexibility and autonomy	*“Flexible working hours”*
		*“Opportunity for remote working”*
		*“Good opportunities to influence one’s own work”*
Personal life and financial conditions	Family life and leisure	*“Family support”*
		*“Other meaningful hobbies”*
		*“Gainful employment is not the most important thing in life”*
	Retirement	*“Voluntary pension insurance”*
		*“I’m not going to stay that long in work”*
	Private economy	*“Thanks to wealth, I can lower my retirement age”*
		*“Far too much debt to pay off still”*
		*“An industry that always has a job”*
Intrinsic work motivation and life orientation	Sustainable work motivation and meaningfulness	*”I will probably have the willingness to work”*
		*“Meaningful work, inspiring tasks”*
		*“The work environment and the interesting discussions that take place there provide stimuli and thoughts also in leisure time”*
	Positive life orientation	*“Take it one day at a time”*
		*“Open mind”*
		*“Attitude to life”*
	Learnt experiences	*“One has coped with difficult situations earlier as well”*
		*“Others can do it”*
Social inclusion and leadership support	Participation and belonging with colleagues	*”Colleagues and a sense of belonging”*
		*“Good work climate”*
	Supportive leadership and organization	*“The trust of the supervisors and the organization”*
		*“That people are noticed even though everything is on business conditions”*
		*“The employer’s appreciation”*
**Categories negatively affecting occupational self-efficacy to continue working until expected retirement age**		
**Categories**	**Sub-categories**	**Quotations**
Insufficient security and work well-being	Changes in the global labor market	*“Replaces with cheap labor force”*
		*“Changes in work tasks or working conditions”*
		*“Too much reorganizing”*
	Uncertainty regarding one’s own resources against challenging job demands	*“To keep up* (*with new programs, etc.*)*”*
		*“Functionality of the information technology [at work]”*
		*“Traveling/long days”*
	Unsatisfying leadership and work community	*“Management does not listen”*
		*“Reduction of opportunities to influence”*
		*“Micromanagement and continuous performance requirements (pressure)”*
		*“In case no one sees and confirms what I do”*
		*“The joy and fellowship that once existed throughout the decision-making chain do no longer exist”*
Declining health	Uncertain health	*”Changes in health, serious illness”*
		*“The unlikelihood of staying healthy until then”*
		*“Health no longer a guarantee”*
	Risk factors for unhealth	*“Poor physical fitness”*
		*“I forget myself”*
		*“Insufficient rest”*
		*“Concerns about illness”*
Consequences of workload	High and increasing workload	*“Unreasonable workload”*
		*“Any increase in workload”*
	Work related stress	*“Continuous stress makes one exhausted”*
		*“Too much stress can cause health problems”*
		*“Nowadays poorer stress management ability”*
Declining prime mover of work	Declining motivation and interest in the work	*“Tedious tasks”*
		*“Motivation stagnates and it’s not possible to change tasks/unit”*
		*“Increasingly chooses to follow from the sidelines (previously active)”*
		*“Would still like to do something completely different before retirement”*
	Contradictions in meaningfulness and values	*“Meaningless tasks”*
		*“Contradictions of values”*
Jurisdiction and societal attitudes	Discrimination	*“The employer usually fires from the oldest end”*
		*“Ageism”*
		*“The Finnish attitude that only younger people are valuable in the labor market”*
		*“Gender* (*i.e., woman*)*”*
	Increased retirement age	*“High retirement age”*
		*“The retirement age seems to be rising all the time”*
Leisure and economic situation	Leisure	*“Prioritization between work and leisure”*
		*“Opportunity for vacation”*
	Income	*“Because of the money I could stay longer too”*
		*“Poor payment for work performed”*
		*“Need for money”*

**Figure 2:**
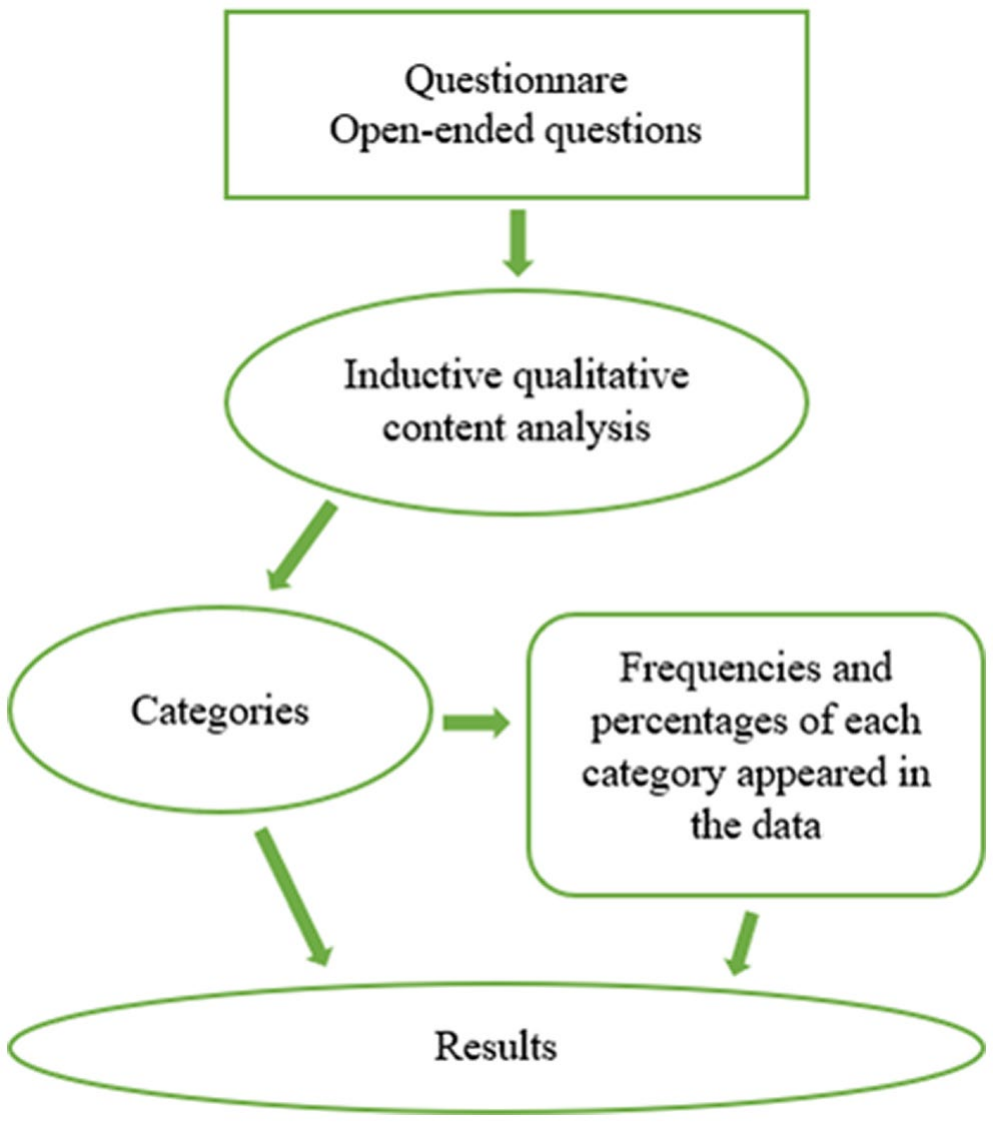
Flowchart of the data analysis and integration procedure.

Next, in the data analysis process, the emerged categories were transformed into quantitative data by counting the number of times each category appeared in the data for each open-ended question (Creswell and Plano Clark, 2018; Kyngäs et al., 2020). This means that every aspect mentioned by each participant, called utterance in this article, was counted within their category. Thereafter, the frequency of the categories was converted into percentages. Counting the frequencies and percentages enabled to examine the distribution of emerged categories and if some of the emerged aspects were prominent. The descriptive statistics were performed using Microsoft Office 2016 Excel. An integration of both the qualitative and the quantitative approach was used in the aim, the data analysis, results, and discussion (Regnault et al., 2018; Kyngäs et al., 2020). The data analysis and integration procedure is described in Figure 2.

Descriptive analyses of the participants’ background characteristics are presented as mean, median, standard deviation and percentage rates in Table 1. Differences between the Swedish- and Finnish-speaking participants were evaluated using Independent Sample *T*-Test and Mann–Whitney *U*-test. Pearson’s Chi-square test and Fisher’s exact test were used for the categorical variables. The data were analyzed using SPSS Statistics 27.0 (IBM Corporation, Armonk, NY, United States).

### 4.3. Ethics approval and consent to participate

This study was approved by the Board for Research Ethics at Åbo Akademi University, Turku, Finland (dated 12 April 2018). The research was carried out in accordance with the ethical guidelines of the Declaration of Helsinki, revised in 2013. All participants received written information about the aim of the study, that participation was anonymous and voluntary, and the right to withdraw from the study at any time. The participants provided informed consent to participate by answering the questionnaire. Aging engineers who wanted to participate gained access to the survey via an attached link in the information letter. Thus, they remained anonymous. No incentives or compensation were given for participation in the study.

## 5. Results

### 5.1. Qualitative analysis

The qualitative content analysis yielded six categories describing what enhances engineers’ self-efficacy to continue working until expected retirement age, and six categories negatively affecting their beliefs. The categories and sub-categories are presented below and illuminated by quotations in Table 2.

#### 5.1.1. Categories positively affecting occupational self-efficacy to continue working

##### 5.1.1.1. Engagement in one’s own health

Satisfactory physical and mental health was emphasized, as well as several activities supporting and promoting one’s own health. The participants stressed the importance of taking care of oneself, including physical and mental health, work-life balance, as well as having control over one’s own life. Seeing colleagues continuing working, despite poorer health than oneself, also enhanced self-efficacy. However, the participants highlighted the importance of being able to adapt the workload to one’s own work ability.

##### 5.1.1.2. Confidence in one’s own competence

Confidence in one’s own competence captures the participants’ acquired knowledge and skills, and continuous ambition and potential to develop in work. The awareness of that they have unique experiences, professional knowledge and skills that are needed and in demand increase their self-efficacy, as well as the feeling of success in work and that they still keep up well with the demands of their work. Henceforth, they want to learn new things, channel their creativity, and seek new challenges in their current or in another work. However, the participants asked for a changed labor market, where experience in combination with knowledge should be demanded of an employee.

##### 5.1.1.3. Healthy work environment

The participants highlighted several different aspects regarding satisfaction with work tasks and work environment; having adequate mental and physical workload, such as suitably challenging tasks and appropriate work tools. In addition, to be able to influence one’s own work, to change work tasks when necessary, work remotely, and have flexibility regarding working hours.

##### 5.1.1.4. Personal life and financial conditions

The participants emphasized the support from their life partners and close family. Meaningful leisure-time activities, time off and holidays are important counterbalances to work. For some engineers, their self-efficacy was enhanced by knowing that they are close to retirement. However, various personal economic conditions also enhance the self-efficacy to continue working. For some participants, having voluntary pension insurance or a good personal economy are opportunities for an earlier retirement, while for others unpaid debts forced their belief to continue working. The continuous need for engineers in the labor market, good economics of the work organization, as well as closeness to the workplace also gave stability that increased the self-efficacy to continue working.

##### 5.1.1.5. Intrinsic work motivation and life orientation

The participants emphasized the meaningfulness of work tasks, that they liked their work itself in a sector that is interesting to them, and that they are still keen to work. Their self-efficacy to continue working depends on whether their work motivation can be maintained, which some easily believed while others were more hesitant about. Furthermore, the participants emphasized the role of their attitude to life and their own willingness to work. They take on the future 1 day at a time and with an open mind. Previous experiences of coping with difficult situations, and of seeing others cope with similar issues and persevere strengthen their self-efficacy to continue working. Moreover, having the point of view that one should not give up also makes them strive further.

##### 5.1.1.6. Social inclusion and leadership support

The importance of participation and a sense of belonging with the colleagues were emphasized; the employee social network, the work community, a good work climate, and a good teamwork. Support from leaders and organization was also highlighted. The participants stressed the importance of honesty, openness, appreciation of and confidence in the employees, and that the organization pays attention to the engineers in an organization that is characterized by business conditions.

#### 5.1.2. Categories negatively affecting occupational self-efficacy to continue working

##### 5.1.2.1. Insufficient security and work well-being

Insufficient security and work well-being capture issues related to a demanding physical, mental and social work environment, and work content. The engineers’ global work environment was stressed as uncertain, and including continuous changes in tasks, conditions, and organization. The participants expressed concerns that the company may move abroad, or that it may not be able to adapt to the rapid development that prevails in this industry. They also conveyed concerns about whether they will be able to learn all the required new things quickly enough, but also that their gained experiences will not be of use in the company. Additionally, challenges in both the physical and psychosocial work environment were stressed; malfunctioning organization, poorly functioning software and short-term solutions, open-plan designed offices, many travel days per year, and a poorer work climate where the previous pleasure at work and good work community have deteriorated. Unsatisfying leadership was also emphasized, including lack of appreciation, respect, and acknowledgement, poor opportunities to influence work, but also high expectations and demands. Likewise, an unclear organization with unclear goals, and a great deal of bureaucracy lowered self-efficacy belief.

##### 5.1.2.2. Declining health

Both current health declines and concerns about future ill-health and diseases negatively affected the participants’ self-efficacy beliefs to continue working. The participants realized that health was no longer a given, and that they are likely to suffer from health problems in the future and/or worsening of current illnesses. They stressed their own personal risk and lifestyle factors for ill-health, such as poor physical fitness, smoking, obesity, insufficient rest, and loneliness. Some participants also expressed reduced quality of their occupational health care service. Additionally, concerns about close relatives’ ill-health decreased the engineers’ own self-efficacy belief to continue working.

##### 5.1.2.3. Consequences of workload

The participants expressed an increasing amount of work to an already high workload. Increased number of work tasks, and increased demands on effectiveness and individual performance cause urgency and stress at work, decreasing self-efficacy belief to continue working. The participants highlighted worries about the perceived too high and continuous mental pressure. They emphasized already existing stress-related health concerns and perceived a poorer stress management ability compared to earlier in life. None of the female participants gave any expressions related to this category.

##### 5.1.2.4. Declining prime mover of work

Declining prime mover of work described decreasing motivation, boredom, monotonous work tasks, and a lack of change and renewal. An increased indifference, or increased negative feelings toward the work, were expressed to negatively affect self-efficacy to continue working. The participants stressed tasks and work content that were perceived meaningless, but also when their own personal values conflicted with those of the organization. Furthermore, the participants pointed out an increased prioritization of time off and hobbies, a desire to do other things in life than work, but also thoughts about working with something else than engineering before retirement.

##### 5.1.2.5. Jurisdiction and societal attitudes

Perceived employer ageism was stated as a barrier to a continued working career. The participants described that the older employees usually were terminated during co-operation negotiations, while the younger ones were allowed to continue working. Engineers highlighted that there is also an existing attitude in the Finnish society that only younger employees are valuable in the labor market. Gender discrimination was also mentioned, however, without any further explanation. Additionally, the increased retirement age in Finland in recent years was also mentioned as lowering the engineers’ self-efficacy to continue working until an expected retirement age.

##### 5.1.2.6. Leisure and economic situation

An increased prioritization of leisure time and opportunities for more time off tempt the participants from working life. Dissatisfaction with salary for the work performed increased the participants’ desire for leaving working life as well. On the other hand, the need for a solid income retained some of the participants in working life. The female participants gave no answers related to this category.

### 5.2. Quantitative analysis

**Figure 3:**
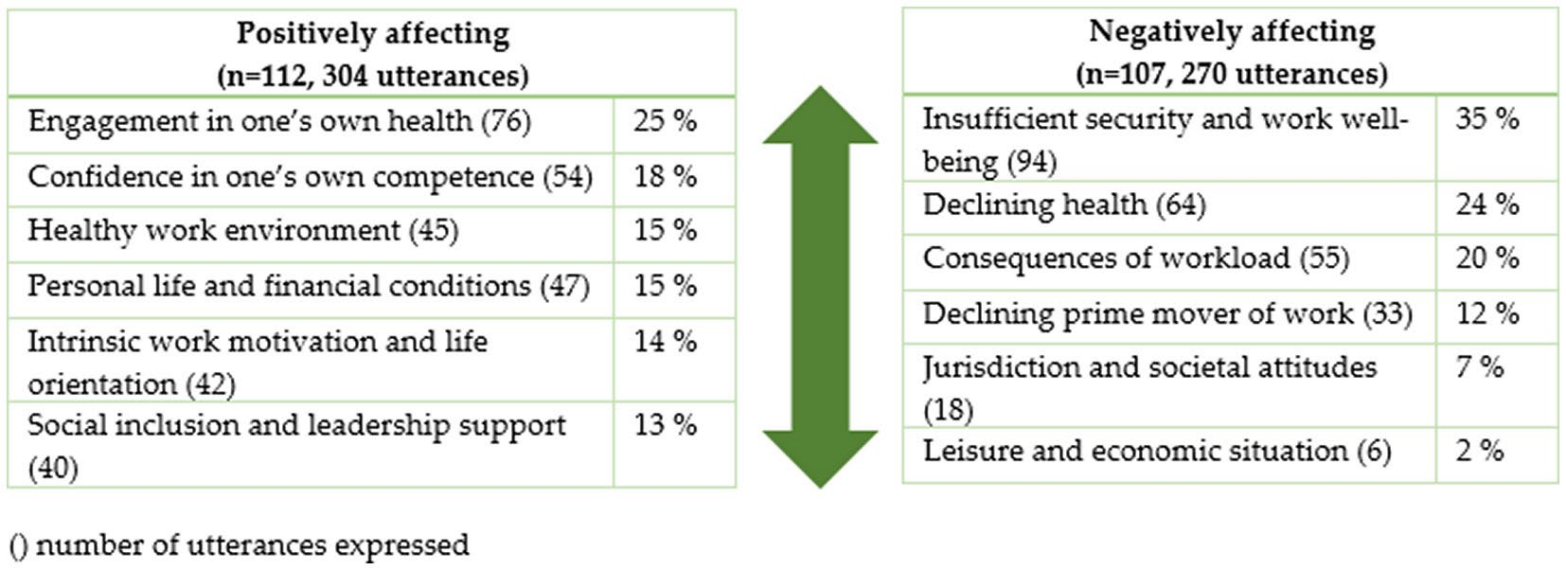
Categories positively and negatively affecting aging engineers’ occupational self-efficacy to continue working until expected retirement age.

There were 304 utterances from 112 participants (90%; 94% of the male engineers and 60% of the female engineers) related to the categories describing what positively affect their occupational self-efficacy belief that they can continue working until expected retirement age. One hundred and seven participants (86%; 86% of the male engineers and 67% of the female engineers) gave 270 utterances that were related to what negatively affect their belief. Typically, the participants gave between one to three responses for each question. The integration of the results from the qualitative analysis into a quantitative analysis is viewed as percentages to describe the relationship between the emerged categories, i.e., which aspects are most often highlighted in the engineers’ answers. The distribution of the responses in frequencies and percentages is presented in Figure 3.

## 6. Discussion

The most important findings show that health aspects are highly important for aging engineers’ occupational self-efficacy to continue working until expected retirement age. Health was either clearly stated by the engineers, or working conditions that affect health and well-being at work. This is *per se* not surprising since health (Carlstedt et al., 2018; Guarnaccia et al., 2018; Laaksonen et al., 2022) and work environmental conditions (Staudinger et al., 2016) are major contributors to long working life. This is also highlighted by Eurofound (2022); the balance between workers’ health, resources, needs and work environmental characteristics are significant for a long working life.

Consistent with the Job Demands-Resources theory (J D-R), personal resources, such as self-efficacy, are important contributors to health and well-being (Bakker and Demerouti, 2017; Galanakis and Tsitouri, 2022). In our study, both the awareness of having, and actively maintaining, a sufficient physical and mental health were important facilitators for the engineers’ occupational self-efficacy. The positive relation between self-efficacy and perceived health, health related behavior, and health related change has been confirmed in previous research (Bandura, 2004; Sheeran et al., 2016; Isa et al., 2017; Galanakis and Tsitouri, 2022). People with high self-efficacy have the incentive to translate health knowledge into health-related outcomes (Bandura, 2004; Sheeran et al., 2016). The participants in our study emphasized several applied health supporting activities that positively affected their self-efficacy to continue working, for example, maintaining their physical fitness and work-life balance. Contrary, they also underscored concerns about already declining health or future ill-health, as well as existing lifestyle risk factors and to not actively cope with health-related threats, which had a negative impact on their self-efficacy. Generally, aging workers are coping with some chronic diseases and health-related limitations (Ilmarinen, 2012; Sundstrup et al., 2017; Eurostat Statistics Explained, 2022), which might challenge their capability and strength. Concerns whether aging workers’ health will be good enough for longer working lives have been emphasized in previous research (Laaksonen et al., 2022). These concerns must be noted, since perceived health is an important predictor of extended working life among aging workers (Nilsson et al., 2016).

According to J D-R theory, adverse job demands require physical or mental effort and cause physiological or psychological costs (Bakker and Demerouti, 2017). The engineers in our study stressed several adverse job demands that negatively affected their self-efficacy. They were dealing with emotional and physical stress reactions related to high workload, high demands, and frequent changes in work description. The engineers stressed concerns about whether these issues will affect their health. When the perceived stress became too much, the engineers’ self-efficacy beliefs sometimes changed into doubts. On one hand, personal resources are assumed to shield the negative impact of adverse job demands on strain; people with high personal resources, such as self-efficacy, have control over their environment and can handle unforeseen events (Bakker and Demerouti, 2017). Individuals with low self-efficacy do not believe that they can control their health habits and will need extensive support and guidance to overcome challenging obstacles and build progressive success through mastery experiences (Bandura, 2004; Bandura, 2012). If workers have already struggled to persevere and faced failures to keep the work up, additional challenges might make them give up trying. Nevertheless, resilient self-efficacy is built on informative learning from failures (Bandura, 2012), of a manageable range. However, even when succeeding in challenging tasks, a great effort required to complete the task might make the person unsure whether one will be able to produce the same effort again (Bandura, 2012). Thus, workers who have successfully coped with very demanding challenges might doubt themselves if they have to meet such challenges again. Ongoing adverse job demands will eventually drain workers’ physical and mental resources and cause health issues (Galanakis and Tsitouri, 2022).

To enhance engineers’ occupational self-efficacy for a full working life, the focus should be on improving the resources they emphasized in our study: healthy work environment, work flexibility and autonomy, competence and learning opportunities, motivation, support from leaders and colleagues, family and leisure. To increase engineers’ self-efficacy and support them in difficult challenges, the issues that they consider the most important must be addressed (Isa et al., 2017). Sufficient individualized support (Eurofound, 2022), enhancing a feeling of success (Bandura, 2004), must be offered to the worker by the workplace leaders as well as the occupational health service. Even though occupational self-efficacy cannot cure the effects of every adverse job demand, high occupational self-efficacy might decrease perceived stress and promote individual and organizational well-being (Onyishi et al., 2018; Tomas, 2021; Galanakis and Tsitouri, 2022), thus support the importance of enhancing occupational self-efficacy.

Private life including partners, close family and leisure were stressed by the engineers as well. Support from partners and family facilitated the engineers’ self-efficacy for a full working life. Meaningful leisure and time-off were important counterbalances to work. However, some engineers emphasized that an increased prioritization of leisure time tempts them from working life. Furthermore, unhealthy relatives decreased their self-efficacy to continue working. Thus, family, relatives and friends affect the workers self-efficacy for a longer career by provided support or social influence (De Vos et al., 2020). This is consistent with previous research; a balance between work and resources at home are supposed to increase self-efficacy (Kossek et al., 2021). A sustainable working life includes a worker’s private life, that might either contribute to or hinder a long working career (De Vos et al., 2020). A sustainable working career is a dynamic process evolving over time. It needs to consider changes within the person and private life, as well as the living context and the work context (De Vos et al., 2020; Hirschi et al., 2020).

Based on the results in our study, to strengthen aging workers’ occupational self-efficacy, along with health issues and fundamental work environmental improvements, the four sources of self-efficacy should be specifically addressed (mastery experiences, vicarious learning, verbal persuasion, and affective states; Bandura, 2012). Questions and themes to be covered through dialogs are: What previous experiences of success in work context can be used to strengthen occupational self-efficacy (mastery experiences)? How have persons comparable to this particular worker done to succeed in similar issues (vicarious learning)? What kind of verbal feedback and encouragement from significant others do this particular worker find helpful to strengthen occupational self-efficacy (verbal persuasion)? How does the particular worker experience, interpret and cope with physical and affective states in demanding situations (affective states)? The workers’ individual needs (Eurofound, 2022) must be in focus, to understand the person, the context, and the life-span perspective (De Vos et al., 2020). These discussions could be preferably conducted in small groups, to share mastery experiences as well as to facilitate vicarious learning and verbal persuasion (Bandura, 2012).

The data material in this study was inductively analyzed, and therefore, the categories that emerged cannot be referred to the separate sources enhancing self-efficacy for engineers (Sheu et al., 2018; Chen et al., 2023). The four sources, that is, mastery experiences, vicarious experience, verbal persuasion, and dealing with emotional and physical reactions (Bandura, 1997; Bandura, 2004; Bandura, 2012), are however, found in the category contents. For example, the participants expressed that their previous experiences (i.e., mastery experiences) as well as seeing other succeed (i.e., vicarious learning) affect their health, work motivation and competence. Social persuasion was seen in our study as support from leaders, management, colleagues, and the close family. A well-functioning and supporting leadership, including attention to and respecting the employees without ageist attitudes increase self-efficacy; this was also found in previous research (Wallin et al., 2022). A note of attention is that gender stereotypes and discrimination was mentioned in our study by female engineers. This also accords with earlier research which showed that female technical workers have experienced discrimination estimated to hamper career development (Salomaa, 2020). Previous research has stressed that women in male-dominated occupations, including engineering, receive less support and are repeatedly excluded from formal and informal social capital and networking opportunities, negatively influencing their work performance as well as their remaining in the engineering workforce (Powell and Sang, 2015). To address this, Powell and Sang (2015) suggest that men must be included in policy initiatives to address this discrimination, because women as under-represented can unconsciously learn the rules of the game and maintain this sad reality. Except for the importance to stop gender stereotypes *per se*, to cover the future need for engineers (Cedefop, 2020) it is of great importance that female engineers remain in the sector.

One additional interesting finding is that the categories that emerged in our study corroborate the recently developed theoretical model SwAge, Sustainable working life for all ages (Nilsson, 2020). SwAge describes factors that determine if people *can* or *want* to participate in working life (Nilsson, 2020), which conspire on different influence levels: individual, organizational and society levels. In this model, factors that determine if people can or want to participate in working life are presented as nine determinant areas concluded under four considerations: personal health in relation to physical and mental work environmental circumstances, private economy, social inclusion and participation in a group, and self-fulfillment by meaningful, stimulating, and creative activities (Nilsson, 2020). Albeit that the SwAge model does not directly relate to the J D-R model, job demands and job resources can be found in the determinant areas and considerations. Neither does the SwAge model specifically discuss self-efficacy; yet mentions self-efficacy as one related factor to manageability, meaningfulness, and comprehensibility, i.e., salutogenesis, see for example Antonovsky (1979), found in the consideration self-fulfillment by meaningful, stimulating, and creative activities (Nilsson, 2021). According to Bandura (2012), “I can” is a statement of efficacy. In SwAge, there are several determinant areas mainly corresponding with “I can” (Nilsson, 2021), that is, the physical and mental work environment, work time, work pace and recovery time, as well as the worker’s knowledge, competence and development of competence. The statement “I want” refers to social work environment, participation, social support and inclusion, as well as work motivation, stimulating and self-crediting tasks, and work satisfaction. Areas related to both “I can” and “I want” are self-rated health and diagnosis, personal finances, and private social environment (Nilsson, 2021). Even though we asked the participants in our study about what affects their confidence in their capabilities that they can continue working, that is, occupational self-efficacy belief, their expressed aspects included all determinant areas and considerations in SwAge, whether the areas and/or considerations cover the statement “I can” or “I want.” To conclude, despite different ways to approach a full working life, the same aspects are important, regardless of whether the workers’ self-efficacy beliefs that they can continue working until expected retirement age, or if the worker can or want to participate in working life, is considered. Furthermore, the findings in this study are consistent with the findings of a previous research that addressed occupational self-efficacy for a full working life among aging home care workers (Wallin et al., 2022). Thus, the findings in this study can with great probability be transferred to aging workers in other occupations as well.

Overall, the results in our study confirm the complexity and multidimensionality of what participating in working life today entails, and the challenges of satisfying needs on several levels to enhance self-efficacy to continue working until expected retirement age. Aspects specific to the workers’ individual needs should be tailored through a person-centered approach and through implementing the guidelines available for enhancing self-efficacy (e.g., Bandura, 2004; Bandura, 2012). Besides, ensuring basic work environmental issues to decrease job demands and increase job resources are still required. Thus, in addition to the worker, the organization, the leaders, the colleagues, and the occupational health services must be involved in this process (Ministry of social affairs and health, n.d.). Through our own behavior and encouraging words we can all contribute to enhancing self-efficacy among our colleagues and leaders.

### 6.1. Methodological considerations

A strength of this study was the use of mixed methods, including both qualitative and quantitative analyses of the research data (Regnault et al., 2018; Kyngäs et al., 2020). To minimize bias, the analyses followed well-reputed recommendations (Graneheim and Lundman, 2004; Graneheim et al., 2017; Creswell and Plano Clark, 2018; Kyngäs et al., 2020). The manifest qualitative analysis provided a deeper knowledge and understanding of the issue (Graneheim and Lundman, 2004; Graneheim et al., 2017; Kyngäs et al., 2020). The number of participants made it possible to quantitatively rank the categories from the qualitative manifest content analysis, based on the number of times each aspect in a category appeared in the data for each open-ended question (Creswell and Plano Clark, 2018; Kyngäs et al., 2020). The quantitatively measured frequencies of utterances gained reliability since the focus of engineers’ experiences was acknowledged (Kyngäs et al., 2020). The integration of the qualitative and the quantitative approaches was used both in the data analysis, results, and discussion (Creswell and Plano Clark, 2018; Regnault et al., 2018; Kyngäs et al., 2020). However, the often short answers in the open-ended questions might have caused some interpretation bias. Furthermore, the cross-sectional design, describing the phenomena at one specific time, limited the ability to obtain causal relationships (Cummings, 2017).

There were twice as many Finnish speakers as Swedish speakers participating in this study and, therefore, despite no significant differences in background factors, socio-cultural biases cannot be excluded. Another limitation in our study was that no background data on formal positions in the company was available. Different formal positions might have influenced the engineers’ self-efficacy beliefs regarding a full working life.

When using a questionnaire there is a risk of high numbers of dropouts. The response rate was 34%, despite three reminders and extended time for recruitment, which might infer a possible outcome bias. Since each company’s local human resource department, not the close managers, provided participants with access to the web-based questionnaire, the survey response might have been negatively affected. Engineers from two companies were excluded before the survey was sent out, because they did not meet the inclusion criteria of the minimum age of 45 years. However, the age limit for aging workers, that is, 45 years and older, was considered representative of aging engineers (Ilmarinen, 2001). Moreover, the companies that were contacted represent workplaces with many engineers and were considered suitable. The response rate of answering each open-ended question was high (90%; *n* = 112 and 86%; *n* = 107), although the open-ended questions were placed last in a large-scaled survey, and so indicated that the participants had an interest in articulating experiences about their self-efficacy to continue working.

The aging engineers were asked what positively and negatively affect their confidence, i.e., self-efficacy, that they can continue working until expected retirement age. How the research questions were formulated might have affected the answers. According to Burrell et al. (2018), questions should be formulated with the verb “can” to answer self-efficacy issues. Therefore, we verified that the survey questions were accurately formulated, to specifically examine self-efficacy. However, it is possible that the participants would have answered differently if a short description of the self-efficacy concept had been given. Since the data material was open-ended questions, it was not possible to ask supplementary questions. Furthermore, this study does not give deeper information about what, on a concrete level, the aging engineers need to enhance their self-efficacy competence, especially regarding the prominent health-related issues.

The participants’ responses were either in Swedish or Finnish. The first author (SW) did the initial coding and translations of Finnish utterances into Swedish. None of the researchers has Finnish as their mother tongue, although two of them master Finnish well. In case of linguistic doubts, a native Finnish speaker was consulted. A strength was that no linguistic misunderstandings appeared in the pilot testing of the open-ended questions. The emerged categories were considered logical and answered the research questions. The findings from our study can likely be transferred to other countries, due to engineering as a global work sector, with employing companies functioning in several countries all over the world (Qadir et al., 2020). Since our findings are in line with those of aging home care workers (Wallin et al., 2022), despite differences in educational level, work context and gender distribution, the results might also be transferable to aging workers in other contexts (Graneheim and Lundman, 2004; Graneheim et al., 2017; Kyngäs et al., 2020).

## 7. Conclusion

The findings revealed the own health and working conditions, which in turn affect their health, as prominent aspects that must be addressed to enhance self-efficacy for a full working life. Furthermore, the engineers emphasized acquired competence and continued competence development as facilitators for self-efficacy, as well as motivation from meaningful tasks. Prioritization of work-life balance and personal finances both tempt the engineers from working life as well as attract or force them to continue working. The findings in our study can aid line leaders in engineering, organizational management, safety staff, occupational health service as well as aging engineers to articulate essential aspects that enhance aging engineers’ occupational self-efficacy. Further research is needed to obtain detailed knowledge how to specifically address the sources of self-efficacy for a full working life.

## Data availability statement

The data is not publicly available but can be requested from the corresponding author after ethical approval to take part of the dataset.

## Ethics statement

The studies involving human participants were reviewed and approved by Board for Research Ethics at Åbo Akademi University, Turku, Finland. The patients/participants provided their written informed consent to participate in this study.

## Author contributions

SW: study design, data collection and analysis, interpreting the analysis, and drafting the manuscript. AF-W and LF: study design, interpreting the analysis, and involved in drafting the manuscript. All authors contributed to the article and approved the submitted version.

## Conflict of interest

The authors declare that the research was conducted in the absence of any commercial or financial relationships that could be construed as a potential conflict of interest.

## Publisher’s note

All claims expressed in this article are solely those of the authors and do not necessarily represent those of their affiliated organizations, or those of the publisher, the editors and the reviewers. Any product that may be evaluated in this article, or claim that may be made by its manufacturer, is not guaranteed or endorsed by the publisher.
